# Railway Spine and Litigation Symptoms

**Published:** 1898-05

**Authors:** W. W. Grant

**Affiliations:** Surgeon Chicago, Rock Island & Pacific Railway, Denver, Col.


### RAILWAY SPINE AND LITIGATION SYMPTOMS.

Presented in the Fourth Annual Meeting of the American Academy of Railway Surgeons at Chicago, Ill., Oct. 6–8, 1897.

BY W. W. GRANT, M.D.
Surgeon Chicago, Rock Island & Pacific Railway, Denver, Col.

From a certain class of spinal symptoms and nervous manifestations following and believed to be typical of railway accidents, is evolved the use of the term "railway spine." The pathologic lesion is not essentially different from similar lesions occurring under other circumstances and conditions, and the term may not be a happy one.

The element of fear enters largely into these histories. There is a distinct appeal to the mental and emotional, and with a certain similarity in clinical histories and results they have been, and are by many at least, believed to be peculiar to railway collisions; so we are justified, as railway surgeons, lawyers and neurologists, in giving them special consideration. We are, perhaps, familiar with those interesting cases following collisions, called often "spinal concussion," in which there is no evidence of a pathologic or anatomic lesion, by any clinical or scientific tests at our command, but in which the patient complains of persistent pain at some point of the spinal column, attended with various nervous manifestations due to the "shock." These cases never recover until there is a definite financial disposition of them. To such cases, an English jurist has applied the term "litigation symptoms."

The teaching of Mr. Erichsen in applying the term "concussion" to all the structures of the spine is too indefinite in history and pathology, and as it does not represent the best thought and opinion of our time, can not be accepted. Concussion of the cord is definite and satisfactory. The cord is so well protected by its environment that concussion is very rare, though believed to be common from railway collision.

Actual lesion of the cord from injury is usually fatal, and when it does happen it is usually attended with direct and manifest injury to some or all of the overlying structures. Meningitis from concussion is equally rare and usually fatal, from whatever cause. It is not with these and the unmistakable injuries of the spine that surgeons, neurologists, railway attorneys and corporations have the most trouble. When the surgeon is confident of a serious lesion, his plain duty to both patient and corporation will often prevent lawsuits. Whether his conclusion is just will depend very much upon the character and completeness of his examination. We can not rely too confidently upon our instruments of scientific precision. The dynamometer, esthesiometer, audiometer, ophthalmoscope, battery, etc., can be weakened by elements of fraud and deception. Mr. Page says plainly that "nothing has struck us as more extraordinary in our experience of railway injuries than that in the examination of them all common sense, the best and surest diagnostic guide, should be so often abandoned and reliance should be rather placed on methods of examination which are of scientific value only when every suspicion of exaggeration or imposture can be put away."

Only a few years ago it was said "no electric test has yet been found which is not rather a test of the credulity of him who trusts it." There is much truth in the statement. The reactions of degeneracy can be proven and the test is often of much value, but only in the hands of a scientific expert, who is equally familiar with the nervous and muscular conditions sought to be elucidated. The manner of the examination is often defective and unwise. I have known these examinations to be conducted in such a way as to suggest to the patient symptoms and ideas that he had not thought of before. An intelligent, shrewd or dishonest individual will utilize them to his own advantage and sometimes to the annoyance of the medical witness and expert.

Not only a full account of the nature of the accident, but the history of the patient prior and subsequent to it is of the greatest importance. The previous condition of health, the vocation, moral status, mental bias, the conduct and character are legitimate matters of surgical inquiry, and often necessary to an intelligent and satisfactory explanation of the case.

Erichsen in his work on spinal concussion uses this language: "An extensive experience in railway compensation cases will probably impress you more with the ingenuity than with the honesty of mankind. A history of deception, practiced on railway companies by alleged sufferers from accidents on their lines, would form a dark spot on the morality of the present generation." Yet, the writings of this distinguished surgeon gave more character to such claims than any other single agency in this generation, though he did not so intend. I believe the statement of Mr. Page true that "molecular disturbance is not necessarily molecular disintegration or pathologic change, and there is no evidence to show that molecular disturbance is, in itself, a grave condition or likely to have evil results." Otherwise it would be something fearful to contemplate the menace to the individual and to the employer from the effects of a jar to the nervous system.

We now know that the serious results from railway collisions are manifested at a very early date after the accident; and in those cases where the symptoms were insignificant or so slight as not to attract the special attention even of the patient and yet at a more remote date, weeks months or years, serious symptoms present themselves, it will generally, I believe, be found that some constitutional infection, such as syphilis or tuberculosis, is the chief factor in the case. And this is as true of the nervous as of the bony and glandular systems.

We hear now of traumatic hysteria; of the neurosis resulting from shock or injury to the nervous system. Obersteiner has recently said, "Where we had hoped to establish a firm anatomic basis, there was only an undemonstrable functional injury of the nervous system, but it is going too far to regard all cases formerly considered as concussions of brain and spinal cord as purely functional." This is, no doubt, true. It is just as true that most of the spinal cases, so commonly called concussions, are not concussions at all; but if any injury really exists, it is contusion or muscular or ligamentous strain. Many of these cases do not recover promptly, but require considerable rest of the parts. They will recover perfectly, however, unless the patient is imbued with the idea that serious results are quite certain to follow "concussion of the spine" from railway accidents.

Under former professional teaching, the opinion, professional and lay, was quite general that the sligher injuries, called usually concussion of the spine, from railway collisions frequently developed at a later period serious symptoms, often with unfortunate results. This is true only exceptionally. But with real concussion of the cord there are generally other unmistakable evidences of injury, and we are enabled to form an intelligent and more satisfactory opinion as to the future.

Concussion of the cord being the exception and not the rule from railway collisions, we ought to leave the unfortunate errors of the past. It is desirable, as soon as possible, to establish the results of railway injuries to the spine upon a pathologic basis. Deferred shock, of which we hear something, is like some of the manifestations of hysteria, emotional or mental. Those cases with slight complaint or manifestations of injury following railway collisions do not, as a rule, at a late day assume a grave form, but they are apt to become litigation cases.

Our experience with such cases, out and in courts of justice, may be commonplace yet beneficial, and I will give one experience:

Three years ago a Denver woman, aged 34, milliner, was on a Rock Island Pullman car at the time of a collision in Kansas. The immediate attendance of railway officials and a local surgeon discovered no passenger injured or complaining. When the lady reached Denver next day, she sent for her physician, who found her with a slight cold only and did not return. In a day or two the company requested me to visit her and report. Recording the result, I found pulse, temperature and pupils normal and no disturbance of motion or sensation general or special; reflexes, normal, but when bending her head and shoulders forward, also on pressure, she complained of pain in the upper dorsal region, but there was no discoloration or evidence of a blow. At time of collision she was asleep, but says her head struck against the headboard, partially doubling her up (which with head toward engine is not improbable). There was total absence of contusion anywhere, but such a position would favor straining or laceration of spinal muscles and ligaments. She came to Colorado eleven years before for supposed lung trouble but had been well ever since. I could discover only a slight cold, resulting probably from delay and absence of fire in a cold season. I gave her a favorable opinion, but from her manner I felt certain she would demand considerable money, so made two or three visits and then had her visit me at my office occasionally for several months, in order to watch developments. To be well prepared, a reputable nerve specialist examined the case at this time, applying all the usual scientific tests and finding no evidence whatever of disease. He asked if she observed any peculiar effects from riding in street-cars and, of course, she answered "yes." Something, I believe, like fear possessed her. She had never before been affected this way, and but for the suggestion of the examiner, I dare say would never have found it out. She demanded $2,000 from the company, was offered $500, brought suit for $15,000 and it was tried in the Federal court two years ago. Nothwithstanding dry cupping and rest for two months following the collision, the spinal pain never ceased and she complained subsequently of headache and sleeplessness at times. The day before the trial she was carefully examined again by the neurologist and myself, and pronounced perfectly healthy. She was so well in her pelvic organs as not even to suggest curetting nor a thought of exploratory section. No symptom is of less value as indicating disease of the cord than spinal pain, yet it is ever present and often with varied and ill-defined nervous manifestations.

A year after the accident she testifies that she is not yet able to conduct her business. I testified that she was as healthy a woman probably as there was in Denver; that if suffering, it was from litigation symptoms and from these would not recover until the case was finally settled; that sometimes such condition of the patient was malingering for the benefit of the money consideration involved. With some claimants the feeling was honestly entertained that they were suffering from the effects of injury, and though they were generally mistaken in this, yet there would be no confession of recovery until the case was definitely settled. There can be no doubt that early settlement in undoubted injury favors earlier recovery.

The neurologist testified that this patient, in his opinion, was not suffering from any disease of the nervous system whatever, but when asked on cross examination to give an opinion on the claimant's own statement of her condition, he said, "She might have hysteria." "And is not hysteria a serious disease?" was instantly asked, and he replied, "Frequently, or sometimes, it is." This statement and that of her own physician, that he could find nothing the matter unless it was hysteria, was absolutely the only medical testimony given in her favor, and the only excuse the jury used, if indeed it needed any, for rendering a verdict of $700 in her favor.

Hysteria as a neurosis, or as an expression of disorder of the generative system, is not a serious condition, and I have been often surprised that even medical experts would, upon the witness stand, use the same language and manner before an ordinary jury, utterly ignorant of the subject, that might be justified before a body of scientific men. This discrimination is an absolute necessity, in the dispensation of justice, to all parties concerned. It is doubtful if a case of importance was ever tried without some medical witness saying something that was unjust to himself and damaging to his cause, and not infrequently because he wished to be supremely scientific and eminently impartial to a body of men incapable of appreciating his meaning.

I meet this woman frequently on the streets of Denver and I have no doubt of her excellent health and her attendance on business.

Within the last year, a Massachusetts lady was on one of the principal trains entering Colorado, when it collided with a snow-bank, causing some delay but, on immediate investigation, no injury to any one Some time after suit was brought againt the road by the lady for deafness due to the collision, and the jury gave her a verdict of over $2,000. My attention was subsequently called to the case, and the railway attorney told me that he accepted the statement of the aurist who attended her only because "it seemed fair." He said no investigation was made as to her previous history, but that her character was a "little shady," and I ventured the opinion that her statement as to her ear trouble probably was also. If an investigation of her past history in Massachusetts had been made, it is not improbable that satisfactory evidence of previous ear trouble could have been obtained.

The medico-legal relations of these railway cases are of peculiar interest to the railway surgeon and the medical expert. The surgical treatment of actual railway injuries is governed by the same general rules and principles which prevail under other circumstances, but always with due regard to the peculiarities of environment. "With the character and manner of conducting these claims we have nothing to do;" but it is as much our duty to understand, if possible, and to appreciate the motives and conduct of these claim patients as it is for the lawyer to prove them. Generally when the clinical history and statements are at variance with those which we should most reasonably expect under ordinary circumstances, we are justified in suspecting ulterior motives, and these are usually of a financial character.

The hope and anticipation of reward consciously, and unconsciously perhaps, influences the course and conduct of individuals in different relations and positions, and this is especially true when compensation for injury and lost time awaits those who are injured in the service of corporations, of which railways afford the most conspicuous if not the most peculiar example.

It is a matter of common observation that the class of patients under consideration are prone to exaggerate every symptom and fear, and if they are silent or their manner unusual upon the statement by the surgeon of a favorable and early recovery, it is safe to assume that a financial problem is under consideration.

How best to meet the actual and varied manifestations of railway injuries and their consequences, near and remote; to separate the imaginary from the real, the fraudulent from the honest claimant, and to properly estimate self-interest and the varied prejudices that enter into consideration *nolens volens* in the endeavor to reach a just solution, is a problem that appeals to the best thought, feeling and judgment of every interested party, and the unremitting labor of a learned profession.—*The Journal.*

